# The efficacy of physiotherapy upon shoulder function following axillary dissection in breast cancer, a randomized controlled study

**DOI:** 10.1186/1471-2407-7-166

**Published:** 2007-08-30

**Authors:** Carien HG Beurskens, Caro JT van Uden, Luc JA Strobbe, Rob AB Oostendorp, Theo Wobbes

**Affiliations:** 1 Centre for Allied Health Research, Dept. of Physiotherapy, Radboud University Nijmegen Medical Centre, P.O. Box 9101, 6500 HB Nijmegen, The Netherlands; 2 Dept. of Surgery, University Hospital Maastricht, P.O. Box 5800, 6202 AZ Maastricht, The Netherlands; 3 Dept. of Surgery, Canisius Wilhelmina Hospital, P.O. Box 9015, 6500 GS Nijmegen, The Netherlands; 4 Centre for Allied Health Research, Dept. of Quality of Care, Radboud University Nijmegen Medical Centre, P.O. Box 9101, 6500 HB Nijmegen, The Netherlands; 5 Dutch Institute of Allied Health Care, P.O. Box 1161, 3800 BD Amersfoort, The Netherlands; 6 Dept. of Surgery, Radboud University Nijmegen Medical Centre, P.O. Box 9101, 6500 HB Nijmegen, The Netherlands

## Abstract

**Background:**

Many patients suffer from severe shoulder complaints after breast cancer surgery and axillary lymph node dissection. Physiotherapy has been clinically observed to improve treatment of these patients. However, it is not a standard treatment regime. The purpose of this study is to investigate the efficacy of physiotherapy treatment of shoulder function, pain and quality of life in patients who have undergone breast cancer surgery and axillary lymph node dissection.

**Methods:**

Thirty patients following breast cancer surgery and axillary lymph node dissection were included in a randomised controlled study. Assessments were made at baseline and after three and six months. The treatment group received standardised physiotherapy treatment of advice and exercises for the arm and shoulder for three months; the control group received a leaflet containing advice and exercises. If necessary soft tissue massage to the surgical scar was applied. Primary outcome variables were amount of pain in the shoulder/arm recorded on the Visual Analogue Scale, and shoulder mobility (flexion, abduction) measured using a digital inclinometer under standardized conditions.

Secondary outcome measures were shoulder disabilities during daily activities, edema, grip strength of both hands and quality of life. The researcher was blinded to treatment allocation.

**Results:**

All thirty patients completed the trial. After three and six months the treatment group showed a significant improvement in shoulder mobility and had significantly less pain than the control group. Quality of life improved significantly, however, handgrip strength and arm volume did not alter significantly.

**Conclusion:**

Physiotherapy reduces pain and improves shoulder function and quality of life following axillary dissection after breast cancer.

**Trial registration:**

ISRCTN31186536

## Background

According to the European Network of Cancer Registration (1999) the incidence of breast cancer in women in the Netherlands is the highest in Europe with figures of 120/100,000. The mean age at which breast cancer is detected is 60 years [1]. Approximately 40% of these women have a metastasis in the axillary lymph nodes, indicating that cancer has possibly spread beyond the breast. The axillary lymph node dissection (ALND) carries a high morbidity, however, as a result of the sentinel node procedure the number of patients with ALND is decreasing. Following surgery with ALND, 73% of women reported restricted shoulder mobility, tightness, edema, pain, numbness of the arm, and limitations in daily life [2,3]. These complaints could be due to tissue and nerve damage. In general, the arm-related complaints usually decrease within three months [4,5]. However, they may also become chronic. The extent of the problem is often underestimated. A study addressing quality of life in patients with breast cancer showed that 74% of the women felt that the ALND had adversely affected their lives [6]. Scar tissue, edema, numbness and possible brachial plexus traction could be the cause. An investigation of recovery of upper limb function after ALND in 76 women by Gosselink et al. showed that three months following surgery, upper limb function is still impaired in a significant number (27%) of patients [7].

Unrelated to breast cancer, there is a high prevalence (7–36%) of musculoskeletal shoulder disorders in the population resulting in considerable pain and disability. Physiotherapy is often the first choice of treatment and has been proven to be effective for these shoulder disorders [8]. However, there is no evidence of the efficacy and effectiveness of physiotherapy for shoulder complaints related to breast cancer and ALND. Kärki et al. deducted from their review that physiotherapy could play an important role in the post-operative treatment of patients with shoulder/arm complaints following breast cancer surgery [5]. Furthermore, Box et al. concluded in their RCT that a postoperative physiotherapeutic protocol is effective in facilitating and maintaining the recovery of shoulder movement [9]. The authors of the review found no evidence showing that the start of early exercises is beneficial. One of the few randomized studies concerning the effect of physiotherapy in patients after breast cancer with ALND showed that physiotherapy leads to a faster functional recovery of the arm [10]. However, follow-up time was short (one to three months). A recent randomized study by Lauridsen et al. (2005), (n = 139), showed a significant improvement of shoulder function after they received team instructed physiotherapy [11]. This study also indicated that patients with breast-conserving therapy showed less severe and less frequent shoulder problems than patients with modified radical mastectomy. Besides the type of surgery the effect of physiotherapy was influenced by adjuvant radiation therapy.

There is no standard referral for physiotherapy following ALND and the disabilities of pain and shoulder dysfunction following this surgery can be severe. The aim therefore of this research is to gain insight into the efficacy of physiotherapy following breast cancer with ALND. The primary measures included shoulder mobility, shoulder and arm function and pain, with quality of life also being assessed.

## Methods

The efficacy of physiotherapy was assessed in a prospective study by comparing two groups of patients who were randomly assigned to a physiotherapy group (exercise therapy) or to a control group. The study was performed from July 2003 to January 2005 and patients enrolled between August 2003 and June 2004. Patients were recruited from the Radboud University Nijmegen Medical Centre (RUNMC) and the Canisius Wilhelmina Hospital (CWZ) Nijmegen in The Netherlands. Patients with breast cancer having to undergo surgery with ALND, were considered eligible for the study and had to meet the following criteria: 18 years of age and older with an ALND, following breast cancer, a Visual Analogue Scale (VAS; 0–10) pain score of 1 or more and moderate shoulder disabilities in daily life (minimal 3 points on a 5 points disability score list). Patients were excluded with a previous contra-lateral breast cancer surgery and insufficient knowledge of the Dutch language to fill in the questionnaires. Informed consent was obtained from all subjects, and the regional medical ethics board approved the study.

Patients were given a subject information sheet by a member of the nursing staff during their hospital stay. Patients who were willing to participate in the trial attended the Department of Physiotherapy for physical assessments two weeks after surgery, which was concurrent to the first outpatient clinic visit to the surgeon. Baseline measurements were assessed and patients who met the inclusion criteria signed an informed consent. Random assignment was done by an independent co-worker of the department into one of the two groups. The treatment group received specific physiotherapy treatment and the control group had no physiotherapy. Concealed randomization was achieved using a computer-generated random list, which was kept by the co-worker. All assessments were done at the RUMC Department of Physiotherapy by a single researcher, who did not participate in the treatment of the patients. The researcher was blinded to the treatment allocation and patients were instructed not to discuss their treatment with the researcher. The researcher made a note after the final assessment, to which group allocation that she thought the patient belonged.

### Intervention

#### Control group

Patients assigned to the control group received a leaflet flyer with advice and exercises for the arm/shoulder for the first weeks following surgery and had no further contact with a physiotherapist.

#### Physiotherapy group

Patients assigned to the treatment group started physiotherapy two weeks following surgery in a private practice of their own choice. The research assistant contacted the individual physiotherapists (n = 15) who had agreed to comply with the treatment regime and supplied them with information regarding the project and treatment guidelines. This information consisted of:

- guidelines with advice and exercises for arm/shoulder, posture correction, coordination exercises, exercises for muscular strength and improvement of general physical condition [12];

- exercises to prevent lymph edema [13];

- instruction for soft tissue massage of the surgical scar if required;

- a form to report the content of the treatment sessions and a 3-point scale to indicate whether the amount of treatment sessions was sufficient.

The total number of treatments was nine (nine being usually covered by the healthcare insurance), once or twice weekly for the first three weeks, and thereafter once a fortnight or less. The total amount of sessions had to be given within three months. Patients were asked to perform home exercises for ten minutes each day.

### Measurements

Demographic data was recorded (age, general health) and as well as data and information about the level of impairment, disability and participation at baseline and after three and six months in both groups.

The primary outcome variables were pain in the shoulder/arm, measured using the VAS score (0 – 10, 0 = no pain; 10 = unbearable pain) and shoulder mobility (flexion [0–180°], abduction [0–180°]), measured by use of a digital inclinometer under standardized conditions.

Secondary outcome measures were disabilities in daily life, measured by the DASH (Disabilities of the Arm, Shoulder and Hand) questionnaire [14] (0 – 100, 0 = no functional problems, 100 = maximal problems), edema (ml), measured in both arms by means of water displacement, grip strength (Kg) of both hands, measured using the hand-held dynamometer and quality of life, as measured by the SIP (Sickness Impact Profile-short version) questionnaire (0 – 68, 0 = good health status; 68 = severe physically disabled) [15]. The total amount of time for each measurement session was approximately 40 minutes, measurements taking place prior to randomization at intake and at three and six months following intake.

### Statistics

Data was analyzed using the SPSS version 12.1. Univariate analysis of variance was used to test differences in outcome variables between the control group and physiotherapy group. Baseline data were entered in the analysis as co-variates. Level of significance was set at 0.05.

## Results

Thirty-six women with breast cancer surgery and ALND were operated during the trial. Six patients did not give informed consent as they were convinced that they needed physiotherapy and did not want to take the risk to be placed in the control group. Thirty-two women with ALND were eligible for inclusion. Two were excluded because they experienced no pain, no shoulder immobility, and no shoulder disabilities. Thirty women, (mean age 55, SD 11, range 34–82) completed the study protocol. In the follow-up period one patient from the control group died before the last assessment. None of the control group received physiotherapy treatment. There were no differences in patient characteristics between both groups at baseline (see Table 1), nor were there any substantial differences in type of adjuvant therapy between the intervention and control group (Table 2). Functional shoulder impairments and pain in the shoulder/arm were reduced significantly after physiotherapy treatment (both p < 0.001) at three months compared with the control group (Tables 3 and 4). In the treatment group the pain decreased on the VAS by 3.4 points in the treatment group (from 4.7 to 1.3), in contrast with a 0.5 point decrease in the control group (from 4.2 to 3.7). Both shoulder flexion and abduction had increased in the intervention group (respectively p = 0.003 and p = 0.005). Shoulder flexion increased in the treatment group by 45 degrees and abduction by 70 degrees versus 11 and 13 degrees respectively in the control group. There was no significant improvement in handgrip strength between both groups (p = 0.08). Volume of the related arm showed no significant difference between both groups at baseline and follow-up (p = 0.88).

**Table 1:** Patient characteristics of intervention group (n = 15) and control group (n = 15) at baseline, no significant differences present between both groups

	Intervention group (n = 15)	Control group (n = 15)
	n	n
Age (mean, SD)	53.7 (SD 13.0)	55.4 (SD 9.3)
Affected side		
*Dominant*	6	7
*Non dominant*	9	8
Pre-existing shoulder complaints		
*None*	13	12
*Rheumatoid Arthritis*	1	3
*Epicondylitis*	1	0
Surgery		
*Breast-conserving and ALND*	3	4
*Mastectomy and ALND*	12	11
Number of extirpated lymph nodes		
*1 – 10 nodes*	2	2
*11 – 21 nodes*	3	1
*>21 nodes*	10	12
Post-surgery complications		
*None*	8	9
*Seroma*	4	3
*Infection*	1	3
*Bleeding*	2	0
Hospital		
*Radboud University MC*	11	10
*CWZ hospital*	4	5

**Table 2:** Adjuvant therapy of intervention group (n = 15) and control group (n = 15)

	Intervention group (n = 15)	Control group (n = 15)
	n	n
None	3	0
Radiation therapy (RT)	0	2
Chemotherapy	2	2
Hormonal therapy	1	1
Radiation therapy and chemotherapy	6	8
Chemotherapy and hormonal therapy	1	1
Radiation and hormonal therapy	1	1
Radio, chemo and hormonal therapy	1	0

**Table 3:** Mean and standard deviation of outcome variables at T0 (baseline), at T1 (after three months), and at T2 (six months)

	**Intervention group**			**Control group**		
	
	(n = 15)			(n = 15)		(*n = 14*)
	
	**T0**	**T1**	**T2**	**T0**	**T1**	**T2**
Outcome	**Mean (SD)**			**Mean (SD)**		

Functional shoulder impairments (1–5)	3.7 (0.6)	1.7 (0.5)	1.4 (0.8)	2.9 (0.6)	3.1 (0.7)	2.4 (0.6)
VAS for pain (0–10)	4.7 (1.6)	1.3 (1.2)	0.9 (1.1)	4.2 (1.8)	3.7 (1.6)	3.2 (1.8)
Handgrip strength (Kg)	26.0 (7.1)	30.0 (6.3)	30.0 (7.0)	24.7 (10.5)	25.7 (11.1)	26.7 (10.1)
Anteflexion shoulder (0–180°)	121 (23.5)	166 (10.1)	171 (13.5)	133 (24.1)	144 (27.0)	153 (22.7)
Abduction shoulder (0–180°)	96.5 (24.0)	167 (15.2)	170 (13.5)	122 (28.9)	135 (38.8)	144 (34.3)
DASH (0–100)	48.6 (18.6)	18.7 (12.7)	14.6 (10.7)	40.5 (20.3)	28.7 (19.1)	21.6 (12.5)
SIP (0–68)	9.1 (6.8)	5.0 (4.5)	4.4 (4.7)	10.5 (9.1)	10.1 (10.8)	8.0 (8.3)
Volume operated arm (ml)	255 (49.1)	261 (55.9)	268 (54.1)	259 (42.9)	263 (50.5)	272 (48.5)

VAS, Visual Analogue Scale; DASH, Disabilities of the Arm, Shoulder and Hand; SIP, Sickness Impact Profile.

**Table 4:** Effect sizes of intervention group compared to control group on determined variables

	**Effect sizes**^a^			
	
	**T0 vs. T1**		**T0 vs. T2**	
	
	Value (95% CI)	Sign (*P*)	Value (95% CI)	Sign (*P*)
Functional impairments (1 – 5)	-1.8 (-2.3 – -1.3)	<0.001	-0.8 (-1.5 – -0.2)	0.018
VAS for pain (0–10)	-2.7 (-3.6 – -1.9)	<0.001	-2.5 (-3.5 – -1.6)	<0.001
Hand grip strength (Kg)	3.1 (-0.4 – 6.6)	0.081	1.4 (-2.4 – 5.2)	0.452
Anteflexion shoulder (0–180°)	24.9 (9.3 – 40.5)	0.003	19.3 (5.7 – 32.8)	0.007
Abduction shoulder (0–180°)	36.7 (12.2 – 61.2)	0.005	29.7 (7.9 – 51.5)	0.010
DASH (0–100)	-13.5 (-24.3 – -2.6)	0.017	-9.0 (-17.2 – -0.8)	0.032
SIP (0–68)	-4.0 (-7.7 – -0.3)	0.035	-2.8 (-6.7 – 1.0)	0.142
Volume operated arm (ml)	1.6 (-20.2 – 23.5)	0.880	-0.6 (-20.7 – 19.4)	0.950

^a ^Effect sizes are calculated as differences between groups at T1 or T2 adjusted for the T0 assessment (entered as covariate in the analysis).

VAS, Visual Analogue Scale; DASH, Disabilities of the Arm, Shoulder and Hand; SIP, Sickness Impact Profile.

Ten patients in the treatment group improved, in participation in social activities and less avoiding heavy work around the house (SIP: p = 0.035). The DASH showed an improvement of shoulder mobility and shoulder/arm disabilities in the treatment group (p = 0.017). For an overview of the effect sizes see Table 4.

Comparison of both groups at six months after intake showed that, except for the SIP, the above-mentioned improvements continued. All physiotherapists who treated patients in the intervention group reported that they had complied with the given instructions and exercises (passive, assisted and active) in nine sessions. Ten (66%) physiotherapists had applied soft tissue massage to the surgical scar, two used (13%) lymph drainage for minimization of edema and four (26%) physiotherapists started with exercises to improve the general physical condition. Eleven (73%) physiotherapists indicated that the number of treatment sessions was sufficient, three (20%) that the number was insufficient and one (7%) that the number was too high. Seven of the eleven physiotherapists indicated that further treatment continuation could be beneficial for the improvement of the general physical condition. The researcher was successfully blinded for treatment allocation of patients; in 60% of the cases treatment allocation was guessed correctly.

## Discussion

This study showed that physiotherapy, which began two weeks after surgery, improved shoulder function and quality of life and reduced shoulder pain in patients with axillary dissection in breast cancer with substantial effect sizes. Handgrip strength showed a positive trend, however this was not markedly impaired postoperatively. The volume of the related arm showed little change with edema commonly occurring at a later stage after surgery. Significant improvement in the psychosocial situation was measured by the SIP. Despite the fact that this questionnaire is not a disease specific instrument, it gives a general idea about how patients cope in daily life. Most patients indicated at intake that they avoided social activities and this improved greatly following therapy.

A sample size calculation was not performed for pain and shoulder mobility in breast cancer patients as insufficient information was available. Outcomes of this study may be used for calculations in a larger effect study, as there are currently no available gold standards.

### Treatment plan

Eleven physiotherapists considered the number of treatment sessions to be sufficient for improvement of shoulder function, however, seven of these physiotherapists reported that further treatment continuation could be beneficial for the improvement of the general physical condition. Nine physiotherapy treatments were opted for, due to the fact that Dutch medical insurance at the time of the study mostly covered the costs for this number of sessions and Harris et al. recommend in their Clinical Practice Guidelines fewer than 12 visits [13]. However, these guidelines are empirical and not evidence based. The literature is not consistent in the amount of physiotherapeutic treatments and the time period. The time period in this study varies from one to three months and the frequency from once to three times a week. Further insight in the optimal treatment frequency and duration is warranted.

While most authors agree that physiotherapy treatment should begin immediately post-operatively, this is not supported by scientific evidence. Other authors suggest starting five to seven days following surgery this having a positive effect on wound healing [16,17]. Research with immediate and delayed onset (3 to 14 days) of exercises showed that benefits of starting early exercises are only marginal [4,16,17]. Lauridsen et al. (2005) showed that, despite patients having postoperative physiotherapy during the first week in hospital, there was compromised shoulder function at seven weeks postoperatively. This improved after 12 sessions of physiotherapy, even when the therapy started after 6 months.

### Ajuvant therapy

The current study size limits conclusions concerning the efficacy of physiotherapy in combination with chemotherapy and radiotherapy. The literature also shows that no conclusion can be made about the best training intensity and duration during chemotherapy and radiation therapy [5]. Future research is needed to examine the effectiveness of these rehabilitation programs. However, adjuvant treatment like radiation seemed to influence the effect on physiotherapy. The subgroup with patients having a breast cancer surgery with ALND and radiation after physiotherapy did not improve their shoulder function significantly. However, Kärki et al. [5] suggest that applying physiotherapy during or after radiation may be of benefit.

### Limitations

Besides the small sample size, a limitation of the study is the short follow-up time of six months. A long-term follow-up will provide further information about the lasting improvement and the occurrence of lymph edema following ALND. The systematic review of Kärki et al. [5] indicated that lymph edema can commence one month to 28 years following surgery. We also found that physiotherapy groups had a significantly improved range of shoulder motion when compared with the control group in all studies. However participating patients in the review had undergone breast cancer surgery with or without ALND. In contrast to the patient groups in the systematic review, the group in our study is homogeneous, i.e. all patients have had breast cancer with ALND.

At this time there is no standard referral for physiotherapy in cases of shoulder/arm related complaints. However, Voogd et al. (2003) found that physiotherapy is often prescribed during follow-up, especially among patients with edema and restricted shoulder function [6]. Up to now there is insufficient evidence of the effectiveness of physiotherapy in this patient group over a longer period of time. Larger studies with at least a 1-year follow-up with relevant outcome measures, such as shoulder function, pain, quality of life and edema are needed. Nevertheless, based on our current findings, we argue that patients with shoulder complaints after ALND should be referred to a physiotherapist. Moreover, a functional shoulder assessment by a physiotherapist at the first outpatient visit two weeks following breast cancer surgery with ALND is also recommended.

## Conclusion

Physiotherapy reduces pain and improves shoulder function and quality of life following axillary dissection in breast cancer.

## Competing interests

The author(s) declare that they have no competing interests.

## Authors' contributions

CB was responsible for the acquisition of the data, participated in the study design, and drafted the manuscript. CvU and RO participated in the design of the study, performed the statistical analysis and interpretation of the data. ThW and LS were responsible for patient inclusion and made substantial contributions to conception and acquisition of the data.

CvU, RO, LS and ThW provided critical edits to this manuscript. All authors read and approved the final manuscript.

## Pre-publication history

The pre-publication history for this paper can be accessed here:


